# Spontaneous Rupture in the Posterior Uterine Wall of an Unscarred Uterus

**DOI:** 10.7759/cureus.112971

**Published:** 2026-07-19

**Authors:** Nadia Marwen, Aymen Khalfaoui, Imen Ketata, Raoudha Rached, Ridha Fatnassi

**Affiliations:** 1 Department of Obstetrics and Gynecology, Faculty of Medicine of Sousse, Ibn El Jazzar Hospital, Kairouan, TUN; 2 Department of Emergency Medicine, Faculty of Medicine of Sousse, Ibn El Jazzar Hospital, Kairouan, TUN; 3 Department of Laboratory Medicine, Faculty of Medicine of Sousse, Ibn El Jazzar Hospital, Kairouan, TUN

**Keywords:** hysterectomy, obstetric emergency, posterior uterine wall, postpartum hemorrhage, unscarred uterus, uterine rupture

## Abstract

Spontaneous rupture of an unscarred uterus is an exceptionally rare but life-threatening obstetric complication. Its diagnosis may be particularly challenging when clinical manifestations are atypical and classical risk factors are absent. We report the case of a 27-year-old gravida 3, para 2 woman with two previous vaginal deliveries and no history of uterine surgery. She presented in spontaneous labor at 39 weeks of gestation. Labor was augmented with oxytocin and resulted in vaginal delivery of a live male neonate weighing 3000 g, with Apgar scores of 9 and 10 at one and five minutes, respectively. During episiotomy repair, abnormal vaginal bleeding was noted despite a well-contracted uterus. Manual exploration of the uterine cavity raised suspicion of a uterine defect, which prompted emergency laparotomy. Surgical exploration revealed a moderate hemoperitoneum and an approximately 8-cm rupture of the left posterior uterine wall of the unscarred uterus extending toward the pouch of Douglas. The uterine defect was initially repaired by primary suturing; however, severe postpartum hemorrhage secondary to refractory uterine atony necessitated a hemostatic hysterectomy. The patient received two units of packed red blood cells and had an uneventful postoperative recovery. This case highlights the importance of maintaining a high index of suspicion for uterine rupture even in unscarred uteri, particularly when unexplained postpartum hemorrhage occurs despite an apparently normal labor and delivery. Early recognition and prompt surgical management are essential to optimize maternal outcomes.

## Introduction

Uterine rupture is a life-threatening obstetric emergency, most commonly observed in women with a history of uterine surgery, particularly cesarean section, where the scar represents a known site of structural weakness and warrants heightened clinical vigilance [1,2]. In contrast, spontaneous rupture of an unscarred uterus is exceedingly rare, with an estimated incidence of approximately one in 17,000 to 20,000 deliveries and an often unpredictable clinical course [3,4]. When it occurs, the consequences can be catastrophic due to the absence of prior warning signs and the potential for atypical presentations. Posterior uterine wall ruptures are especially challenging, as this location is less accessible to clinical examination and may remain undetected until serious complications develop [5].

Although rare, uterine rupture is associated with significant maternal and fetal morbidity and mortality. Maternal complications include massive hemorrhage, hypovolemic shock, hysterectomy, and, in severe cases, maternal death [5]. Fetal complications range from acute fetal distress and hypoxic injury to perinatal death. Several risk factors for rupture of an unscarred uterus have been reported, including uterine malformations, grand multiparity, abnormal placentation, obstructed labor, fetal macrosomia, multiple gestation, previous uterine curettage, and the use of uterotonic agents [6]. Nevertheless, spontaneous rupture may occasionally occur in the absence of major predisposing factors, making diagnosis particularly challenging [3].

This case is notable because it occurred in a young multiparous woman with an unscarred uterus and no history of uterine surgery, whose pregnancy and labor were apparently uncomplicated. Although multiparity and oxytocin augmentation were present, no major predisposing factor for uterine rupture was identified. Furthermore, the rupture involved the posterior uterine wall and remained clinically unrecognized until the postpartum period, when abnormal bleeding during episiotomy repair prompted further investigation. This delayed presentation highlights the diagnostic challenges of posterior uterine rupture and underscores the importance of considering this rare condition even after an apparently uncomplicated vaginal delivery.

## Case presentation

We report the case of a 27-year-old gravida 3, para 2 woman with a history of two previous full-term vaginal deliveries and two living children. She had no significant medical or surgical history, and her current pregnancy had been regularly followed without complications.

The patient was admitted to our department in spontaneous labor at 39 weeks of gestation. On admission, she was hemodynamically stable, with a blood pressure of 110/70 mmHg, a heart rate of 85 beats/min, and a normal body temperature. Abdominal and obstetric examination revealed regular uterine contractions, cervical dilatation of 6 cm, intact membranes, and a cephalic presentation with a non-engaged fetal head.

Ultrasound examination performed on admission demonstrated a singleton fetus in cephalic presentation, an anterior placenta, a normal amniotic fluid index, and an estimated fetal weight of 3100 g. Fetal heart rate monitoring remained reassuring throughout labor, with no evidence of fetal distress. Initial laboratory investigations showed a hemoglobin level of 12.5 g/dL.

Labor was augmented with oxytocin, administered at 5 IU in 500 mL of normal saline. The infusion was initiated at approximately 2 mIU/min and progressively titrated over approximately four hours until an adequate uterine contraction pattern was achieved, resulting in three contractions every 10 minutes. The first stage of labor lasted approximately nine hours, while the second stage lasted 45 minutes. Labor progressed normally to full cervical dilatation, resulting in vaginal delivery of a live male neonate weighing 3000 g. Apgar scores were 9 and 10 at one and five minutes, respectively.

Immediately after placental delivery and during episiotomy repair, abnormal dark vaginal bleeding was observed despite a well-contracted uterus. The estimated blood loss was approximately 500 mL, collected in a graduated container. This unexpected finding prompted manual exploration of the uterine cavity, which revealed a suspected defect in the posterior uterine wall. The diagnosis was therefore considered immediately postpartum, and the patient was promptly transferred to the operating room for emergency laparotomy.

**Figure 1:**
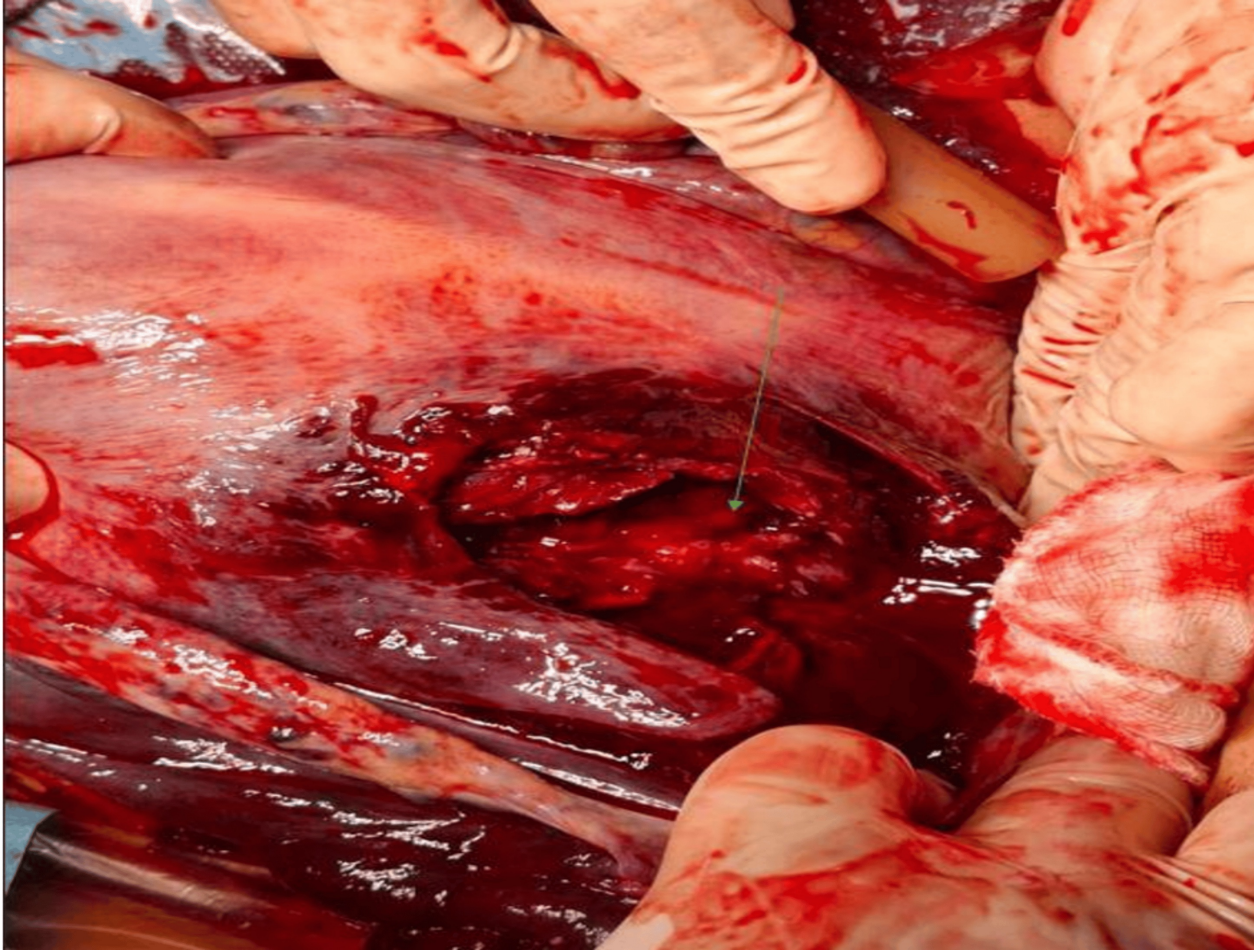
Intraoperative view showing an approximately 8-cm rupture of the left posterior uterine wall (green arrow) extending toward the pouch of Douglas associated with moderate hemoperitoneum

Surgical exploration revealed a moderate hemoperitoneum and an approximately 8-cm rupture of the left posterior uterine wall extending inferiorly toward the pouch of Douglas (Figure 1). The uterine defect was repaired by primary repair of the uterine defect with the intention of preserving the uterus and future fertility.

Following repair, the patient developed severe uterine atony associated with persistent postpartum hemorrhage. The uterine repair remained intact; however, bleeding continued despite administration of uterotonic agents and supportive resuscitative measures. Approximately 90 minutes after the diagnosis of uterine rupture, and despite attempts at uterine preservation, a hemostatic hysterectomy was performed as a life-saving procedure because of ongoing hemorrhage and the risk of further hemodynamic deterioration.

Two units of packed red blood cells were transfused. Hemoglobin decreased from 12.5 g/dL on admission to 7.0 g/dL following the hemorrhage and increased to 9.5 g/dL after transfusion.

The postoperative course was favorable, with progressive hemodynamic stabilization. The patient was monitored in the intensive care unit for three days and was subsequently transferred to the maternity ward. She was discharged home in good clinical condition on postpartum day 4.

To improve readability and provide a clear overview of the clinical course, Table 1 summarizes the labor course, diagnostic process, surgical management, transfusion requirements, decision to perform hysterectomy, and postoperative outcome.

**Table 1:** Timeline of clinical events ICU: intensive care unit

Time/event	Clinical findings and management
Admission at 39 weeks	Hemodynamically stable; cervix 6 cm dilated; cephalic presentation; intact membranes
Labor augmentation	Oxytocin 5 IU in 500 mL normal saline for approximately four hours
Labor progression	Adequate contraction pattern (three contractions/10 minutes); first stage nine hours; second stage 45 minutes
Delivery	Vaginal delivery of a live male neonate weighing 3000 g; Apgar scores 9 and 10 at one and five minutes
Immediate postpartum period	Dark vaginal bleeding despite a well-contracted uterus
Diagnostic evaluation	Manual exploration of the uterine cavity suggested a posterior uterine wall defect
Emergency laparotomy	Moderate hemoperitoneum and an 8-cm rupture of the left posterior uterine wall extending toward the pouch of Douglas
Initial surgical management	Primary repair of the uterine defect
Approximately 90 minutes later	Persistent hemorrhage due to severe uterine atony despite medical treatment
Definitive treatment	Hemostatic hysterectomy
Blood transfusion	Two units of packed red blood cells
ICU stay	Three days
Discharge	Postpartum day 4 in good clinical condition

During the postoperative period, the patient received detailed counseling regarding the diagnosis, the surgical management undertaken, and the implications of hysterectomy for future fertility. Psychological support was also provided to assist her in coping with the emotional impact of this unexpected obstetric complication.

## Discussion

Uterine rupture is defined as a discontinuity of the uterine wall occurring during pregnancy or labor, with approximately 75% of cases reported during labor [2]. Uterine rupture is classically categorized into two types: complete uterine rupture, which involves a full-thickness tear of the uterine wall (endometrium, myometrium, and serosa), resulting in direct communication between the uterine and peritoneal cavities, and incomplete uterine rupture (uterine dehiscence), which involves disruption of the endometrium and myometrium while preserving the integrity of the uterine serosa [7].

Uterine rupture in an unscarred uterus is a very rare complication in developed countries but remains more frequent in developing nations. This disparity may reflect differences in socioeconomic conditions and access to adequate prenatal care. In unscarred uteri, the incidence of uterine rupture is estimated to range from one in 17,000 to one in 20,000 deliveries [8].

Uterine rupture is generally classified as either traumatic or spontaneous. Traumatic uterine rupture may result from external abdominal trauma or iatrogenic causes, including intrauterine manipulations or excessive fundal pressure during labor. In contrast, spontaneous uterine rupture occurs in the absence of trauma and often presents suddenly, making it a particularly serious obstetric emergency [1].

Spontaneous rupture of an unscarred uterus during pregnancy is exceptionally rare but potentially catastrophic. Several risk factors have been identified in the literature, including uterine anomalies (such as bicornuate or septate uterus), grand multiparity, uterine overdistension (due to macrosomia or multiple gestation), abnormal placentation, previous uterine curettage, obstructed labor, instrumental delivery, and the use of uterotonic agents, particularly oxytocin [7]. Oxytocin, commonly used for labor induction or augmentation, has been associated with an increased risk of uterine rupture when excessive uterine activity occurs.

In our patient, no major predisposing factor for uterine rupture was identified. She was multiparous and received oxytocin augmentation during labor. However, no evidence of uterine hyperstimulation was documented. Although oxytocin exposure may have contributed to increased uterine contractile activity, its exact role in the rupture cannot be established. Labor was monitored through regular assessment of uterine contractions, cervical dilatation, fetal descent, and fetal heart rate monitoring, with no signs of fetal distress or labor dystocia. This case highlights the importance of careful monitoring during labor, even in women with unscarred uteri and apparently uncomplicated pregnancies [9].

Several authors have reported spontaneous uterine rupture in the absence of identifiable risk factors. Chang et al. [10] reported cases occurring without recognized predisposing conditions. More recent literature suggests that subtle uterine abnormalities, including uterine diverticula, arteriovenous malformations, and endometriosis, may contribute to these unexplained ruptures [11,12]. Furthermore, histopathological alterations of the lower uterine segment have been proposed as possible mechanisms underlying uterine rupture. Liu et al. [13] described muscle fiber damage and structural weakening of the lower uterine segment that may predispose to rupture during labor.

Uterine rupture most frequently occurs in the lower uterine segment, particularly during labor, as this area undergoes significant thinning and mechanical stress from the descending fetal presenting part. In contrast, when rupture occurs outside labor, it more commonly involves the uterine body [10]. Posterior uterine ruptures are rare and are often associated with specific risk factors such as previous uterine surgery, abnormal placentation, or uterine anomalies [12,13]. In our case, the rupture involved the posterior uterine wall, an unusual location in the absence of recognized predisposing factors. This atypical presentation suggests a possible subtle underlying myometrial weakness that may not be clinically or radiologically apparent [8].

Our case shares several features with previously reported cases of spontaneous rupture of an unscarred uterus, including the rarity of the condition and the diagnostic challenge it presents. Similar cases reported in the literature were frequently diagnosed following acute abdominal pain, fetal distress, maternal hemodynamic instability, or intrapartum deterioration [10,13]. In contrast, our patient remained clinically stable throughout labor and delivery. The rupture was only suspected postpartum because of unexplained vaginal bleeding despite a well-contracted uterus. Furthermore, the posterior location of the rupture and the absence of major predisposing factors such as a uterine scar, uterine anomaly, or abnormal placentation make this presentation particularly unusual [8,12,13].

This case highlights an important clinical lesson: posterior uterine rupture may remain clinically silent during labor and delivery [13]. Unexplained postpartum bleeding despite a well-contracted uterus and the absence of an obvious source of hemorrhage should prompt consideration of uterine rupture and immediate evaluation. Early recognition of these atypical findings and timely surgical exploration are essential to reduce maternal morbidity and improve outcomes [2,11]. Although primary repair of the uterine defect was initially attempted in an effort to preserve fertility, persistent postpartum hemorrhage secondary to severe uterine atony ultimately required life-saving hemostatic hysterectomy.

## Conclusions

Spontaneous rupture of an unscarred uterus is a rare but potentially life-threatening obstetric emergency. This case illustrates an unusual posterior uterine rupture that remained clinically silent during labor and was diagnosed only in the postpartum period following an apparently uncomplicated vaginal delivery. Unexplained postpartum bleeding despite a well-contracted uterus should prompt consideration of uterine rupture and immediate evaluation. In this patient, primary repair of the uterine defect was initially attempted to preserve fertility; however, persistent hemorrhage associated with severe uterine atony ultimately required hemostatic hysterectomy. Early recognition and timely surgical intervention are essential to reduce maternal morbidity. As a single case report, this observation cannot establish causality or management recommendations but highlights the importance of maintaining a high index of suspicion for atypical presentations of uterine rupture in women with an unscarred uterus.
